# On the Diagnostic Value of the Absence of Chlorides from the Urine

**Published:** 1854-06

**Authors:** 


### RECORD OF MEDICAL SCIENCE.

*On the Diagnostic value of the absence of Chlorides from the Urine.* —Simon and Redtenbacher first stated that chloride of sodium, a salt always present in healthy urine, was absent from that fluid during the onward progress of pneumonia, and returned to it when absorption of the exudation was about to commence. This statement was confirmed by Dr. Beale of London, who, in the 35th vol. of the Transactions of the Medico-Chirurgical Society of London, furthered our knowledge regarding it by additional valuable researches. My attention was directed to this remarkable fact during the present session by Dr. Robert Cartwright, a gentleman attending the Clinical Wards of the Infirmary, who informed me that he had seen it occasionally of great service in a diagnostic point of view, in the clinical wards of Professor Oppolzer at Vienna. It so happened that a man, John M'Donald, æt. 25, had just been admitted laboring under well marked simple pneumonia at the apex of the right lung. He was a laborer, who had enjoyed perfect health until two days before admission, when, on being exposed to wet and cold working at drains, he was seized with shivering followed by fever, and the usual symptoms and signs of pneumonia. On adding a drop of nitric acid to some of his urine in a test tube, and then dropping into it a little solution of the nitrate of silver, the fluid remained clear, although so great is the delicacy of this test, that a white cloudy precipitate is at once formed, if a very minute quantity of the chloride of sodium be present. It was on the fourth day of the disease that the observation was first made, and the chlorides remained absent during the fifth and sixth days, during which period the disease extended from above downwards, until it occupied the upper two-thirds of the right lung. On the seventh day a slight haze was observed in the urine, indicating that the salt was returning to that fluid, and the man expressed himself as being much better. On this day there was great dulness on percussion, all crepitation had ceased, the breathing was tubular with bronchophony. On the eighth day slight returning crepitation was audible, the dulness had diminished, but the urine, owing to some accident before the visit, had been thrown away. On the ninth day, however, the chlorides were abundant in that fluid, together with lithates, loud crepitation was now universal throughout the lung, and the dulness had nearly disappeared. From this time the man made a rapid recovery, never having been bled, and was discharged quite well on the sixteenth day.

I now requested Mr. Seymour, one of the clinical clerks, to test the urine of all the patients in the ward, and others who might subsequently be admitted, which he did, and thus collected a large number of observations, the results of which I shall allude to immediately. In the mean time another case entered, which seemed to point out the value of this test in a diagnostic point of view. It was that of a man, Donaldson, æt. 26, laboring under typhus fever, in whom the disease ran its usual course to the tenth day, when chlorides were demonstrated in it. On the 11th day, however, pulmonary symptoms came on, and the chlorides were entirely absent from the urine. This led me to make, with the clinical class, a careful examination of the chest, when all the signs of pneumonia were detected in the lower half of the right lung. On the fourteenth day the chlorides reappeared, the pneumonic signs diminished (?) and the fever ceased with a critical sweat.

The third case was even more satisfactory in proving the moment of commencing and departing pneumonia by testing the urine for chloride of sodium. A man called David Murray, æt. 43, entered with pneumonia of the lower two-thirds of the right lung. No consistent account could be obtained from him as to when the disease commenced, and it was impossible, therefore, to determine whether the coarse crepitation which was audible over the inflamed lung was the advancing or returning crepitation. But the chlorides were absent from the urine, which indicated that the disease was advancing. The following day complete consolidation had occurred, with dry tubercular breathing and absence of crepitation, and a minute quantity of the chlorides was found in the urine. The patient, however, instead of getting better showed no improvement, and the next day the chlorides had again disappeared, indicating extension of the pneumonia. On the evening of this day he was seized with acute meningitis of which he died. On dissection, in addition to universal cerebral meningitis, the whole of the right lung presented the usual characters of grey hepatization.

These cases serve to point out a remarkable connection between the absence of chlorides from the urine and the onward progress of pneumonia. I forbear from offering any opinion as to the theories which have or may be advanced on this subject. The fact requires to be more extensively investigated clinically than has yet been done to test its value.

Mr. Seymour tested with great care, and at repeated times, the urine of upwards of fifty other cases in the wards, embracing a great variety of disease. He found the chlorides absent in one case of phthisis, with intercurrent pneumonia, but in no other. They were also absent in one case of peritonitis, and in all the cases of small-pox. Further investigation will probably discover these salts to be absent in other diseases, which, although it may diminish the importance of the sign as distinctive of pneumonia, leaves unaffected its value as pointing out the onward progress of that disease.

I need only now allude to one other point, viz., that if any phosphates exist in the urine, nitrate of silver throws down a faint sediment, which, although it cannot be mistaken for the precipitate of chlorides in healthy urine, may be confounded with the appearance it presents when small in amount. In such a case the action of ammonia, by dissolving the chlorides, is at once distinctive.—*Edin. Monthly Journ. of Med. Science.*

